# Supplementary material for: Distinguishing the sources of silica nanoparticles by dual isotopic fingerprinting and machine learning
Source: Nat Commun. 2019 Apr 8;10:1620. doi: 10.1038/s41467-019-09629-5 (PMC6453897; doi:10.1038/s41467-019-09629-5)
Supplement: Supplementary file 1 — Supplementary Information [file 41467_2019_9629_MOESM1_ESM.pdf]

SUPPLEMENTARY INFORMATION

for

Distinguishing the sources of silica nanoparticles by dual isotopic fingerprinting and machine learning

Yang et al.

Content

1. Supplementary Methods
2. Supplementary Discussion
3. Supplementary Tables
4. Supplementary Figures
5. Supplementary References

1. Supplementary Methods

1.1 Chemicals and Reagents

The Si and O isotope standard NIST SRM-8546 was purchased from the National Institute of Standards and Technology (Gaithersburg, MD). The secondary Si isotope standard IRMM-017 was bought from the Institute for Reference Materials and Measurements (GEEL, Belgium). The element calibration standard solution was bought from Agilent (Santa Clara, CA). Nitric acid (65%) was bought from Merck (Darmstadt, Germany). Hydrochloric acid (37.5%, MOS) was purchased from Beijing Chemicals Works (Beijing, China). Sodium hydroxide and Hydrogen peroxide were purchased from Sinopharm Chemical Reagent Co. (Beijing, China). Ultrapure water (18.3 MΩ·cm) used throughout the experiments was produced from a Milli-Q Gradient system (Millipore, Bedford).

1.2 Collection of real consumer products

Four types of toothpaste (TP) samples, three types of inorganic filter membrane (IFM) samples, and one nanoquartz coating (NQC) sample were purchased from different manufacturers or suppliers. The detailed information about the manufacturers or suppliers is given in Supplementary Table 1 and 2.

1.3 Extraction of SiO_2 NPs from real products

To extract SiO_2 particles from real samples, 0.2 g of samples was introduced into a Teflon digestion vessel containing concentrated HNO_3 (4.0 mL) and H_2O_2 (4.0 mL). Then, the vessel was sealed and digested in a microwave system. The mixture was irradiated at 150 ℃ (800 W) for 10 min followed by 180 ℃ (1600 W) for 20 min. After cooling, the mixture was transferred to a 50 mL centrifuge tube and washed with ultrapure water for three times. Finally, the residues were heated at 100 ℃ to dryness. The purity of the extracts was checked by using XRF and ICP-MS.

1.4 Construction of pseudo-samples used in the model study

In order to obtain an unbiased classifier, balanced data samples for the three to five classes of sources should be ensured for the machine learning procedure. Therefore, a randomly

sampling procedure was conducted to generate pseudo-samples for two classes (ND and NQ) with limited experimental data from the literature data. For each class, the δ^{30}Si and δ^{18}O values of pseudo-samples were randomly drawn from the literature reported data[1-12] that followed a normal distribution (1.41 ± 0.26‰ and 31.99 ± 5.45‰ for δ^{30}Si and δ^{18}O of ND, respectively; -0.26 ± 0.42‰ and 10.62 ± 0.66‰ for δ^{30}Si and δ^{18}O of NQ, respectively). The combination of the experimental data with the literature data can also enhance the representiveness of the data. The sampled data listed in the Supplementary Table 3-5 are labeled by asterisks.

2. Supplementary Discussion

2.1 Explanation for the wide δ^{30}Si range of EF

The EF showed a much wider δ^{30}Si range than ES and EP (Fig. 2a). This phenomenon might be related to the large isotopic enrichment factor and highly uncertain relative fraction reacted during the flame pyrolysis of $SiCl_4$ (reaction **10** in Fig. 3). The wide δ^{30}Si range of EF produced from the same precursor ($SiCl_4$) also suggested that the reaction **10** was the key step to cause a large Si isotope variation in EF. The lower boundary of δ^{30}Si range of EF (-5.74‰) approached to the most negative δ^{30}Si value ever found in the terrestrial system[3], suggesting that the reaction **10** had a large isotopic enrichment factor. Furthermore, in a kinetically controlled system with negligible backward reaction, the degree of isotope fractionation is dependent on the relative fraction reacted[13]. We note that the wide δ^{30}Si range of EF was not only caused by the variation among different manufacturers. As shown in Fig. 4c-d, even in EF products from the same manufacturer, the δ^{30}Si also showed large variations. The possible reason is that the flame pyrolysis of $SiCl_4$ is a violent reaction and the reaction yield is rather unstable. The products obtained at different yields may have different isotopic fingerprints due to the different relative fractions reacted. In addition, the unreacted reactants may also have different isotopic fingerprints, and they are normally reused in the industrial production, which may obliterate the Si isotope fractionation in the end product and bring in more uncertainties to the isotopic fingerprints of products.

2.2 Isotope fractionation during the EP production in different manufacturers

We have investigated the Si and O isotope fractionation during the manufacturing process of precipitated SiO_2 NPs (EP). The Si and O isotopic compositions of EP and its raw material NQ from three different manufacturers were analyzed (Supplementary Fig. 8). For O isotope, all product EPs were enriched in ^{18}O relative to the raw materials, probably due to the introduction of external ^{18}O-enriched sources (e.g., NaOH) during the manufacturing process (see Supplementary Table 8, reaction **5**). It should be noted that the industrial synthetic routes to NaOH are rather complex, which may cause the O isotopic composition of industrial NaOH highly uncertain. This could explain why the EP showed a relatively wide δ^{18}O range. For Si

isotope, the variations in δ^{30}Si of EP were not as large as those of EF (Fig. 2a), suggesting that the precipitation method did not cause large Si isotope fractionation. Specifically, the manufacturing process in different manufacturers caused different degrees of Si isotope fractionation, suggesting that the Si isotope fractionation was also affected by the manufacturing conditions. For EP from THSL, no significant Si isotope fractionation between products and raw materials was observed. For EP from WFSJ, the Si in products was enriched in light isotope, while that from SGCT were slightly enriched in heavy isotope. The explanation for these phenomena was not very clear yet, while the different Si isotope fractionation degree may provide another manner to distinguish the manufacturers of EP.

3. Supplementary Tables

Supplementary Table 1. Sample information, Si and O isotopic compositions, and O/Si ratios ($R_{O/Si}$) of all samples used in this study.

No.	Sample[a]	Type	Model/Information	δ^{29}Si (‰)	SD	δ^{30}Si (‰)	SD	*n*	δ^{18}O (‰)	SD	*n*	$R_{O/Si}$	SD	*n*
1	WFSJ-P1	Precipitated silica	650 nm, hydrophilic	-0.36	0.08	-0.72	0.06	2	19.0	0.05	5	2.19	0.64	5
2	WFSJ-P2	Precipitated silica	650 nm, hydrophobic	-0.53	0.05	-1.02	0.14	3	18.7	0.05	5	3.27	0.57	6
3	WFSJ-P3	Precipitated silica	2500 nm, hydrophilic	-0.54	0.03	-0.96	0.05	2	21.4	0.01	5	2.86	0.31	6
4	WFSJ-Q	Quartz	-	-0.04	0.12	-0.30	0.01	2	11.8	0.03	5	-	-	-
5	TCTS-P	Precipitated silica	SP1, 20 nm	-0.18	0.05	-0.25	0.03	2	25.9	0.02	5	2.95	0.33	7
6	BJDK-P1	Precipitated silica	30 nm	-0.19	0.05	-0.28	0.09	3	20.3	0.06	5	2.90	0.13	6
7	BJDK-P2	Precipitated silica	100 nm	-0.45	0.10	-0.75	0.10	2	20.0	0.04	5	2.03	0.35	6
8	BJDK-P3	Precipitated silica	500 nm	-0.45	0.02	-0.75	0.08	3	18.2	0.04	5	2.09	0.45	7
9	XCJR-P1	Precipitated silica	VK-SP15, 15 nm	-0.42	0.04	-0.73	0.10	3	18.9	0.04	5	2.76	0.43	7
10	XCJR-P2	Precipitated silica	VK-SP20, 20 nm	-0.32	0.03	-0.64	0.13	3	18.7	0.02	5	2.53	0.50	5
11	XCJR-P3	Precipitated silica	VK-SP30, 30 nm	-0.37	0.01	-0.69	0.09	3	18.6	0.05	5	2.25	0.13	5
12	XCJR-P4	Precipitated silica	VK-SP50, 50 nm	-0.41	0.06	-0.62	0.01	2	21.0	0.01	5	2.51	0.46	5
13	SGCT-F1	Fumed silica	20 nm, hydrophilic	-2.78	0.03	-5.19	0.17	3	21.4	0.03	5	2.39	0.21	9
14	SGCT-F2	Fumed silica	20 nm, hydrophobic	-1.41	0.03	-2.73	0.11	3	22.1	0.04	5	2.62	0.14	4
15	SGCT-F3	Fumed silica	40 nm, hydrophilic	-3.04	0.02	-5.74	0.12	3	21.6	0.04	5	2.65	0.21	8
16	SGCT-F4	Fumed silica	40 nm, hydrophobic	-1.55	0.08	-2.97	0.14	3	22.4	0.03	5	2.45	0.60	8
17	SGCT-F5	Fumed silica	50 nm, hydrophilic	-1.26	0.04	-2.39	0.10	3	22.3	0.07	5	2.21	0.19	5
18	SGCT-F6	Fumed silica	100 nm, hydrophilic	-1.39	0.08	-2.73	0.16	3	17.3	0.08	5	2.31	0.30	4
19	SGCT-S1	Sol-gel silica	1500 nm	-0.21	0.05	-0.37	0.15	3	16.9	0.04	5	2.23	0.34	8
20	SGCT-S2	Sol-gel silica	2000 nm	-0.18	0.04	-0.40	0.09	3	18.4	0.05	5	2.40	0.44	12
21	SGCT-S3	Sol-gel silica	3500 nm	-0.30	0.04	-0.75	0.14	3	18.9	0.03	5	2.44	0.36	12
22	SGCT-P1	Precipitated silica	120 nm	-0.03	0.03	-0.17	0.02	3	20.3	0.03	5	2.28	0.31	8
23	SGCT-P2	Precipitated silica	200 nm	-0.10	0.05	-0.24	0.14	3	20.5	0.03	5	2.02	0.08	4

24	SGCT-P3	Precipitated silica	325 nm	-0.12	0.05	-0.12	0.17	3	20.8	0.05	5	2.08	0.18	4
25	SGCT-P4	Precipitated silica	500 nm	-0.15	0.03	-0.19	0.17	3	16.4	0.04	5	2.09	0.18	5
26	SGCT-P5	Precipitated silica	1250 nm	-0.03	0.03	-0.16	0.25	3	17.1	0.04	5	2.65	0.28	8
27	SGCT-P6	Precipitated silica	1500 nm	0	0.12	-0.09	0.16	3	20.5	0.02	5	2.40	0.40	10
28	SGCT-P7	Precipitated silica	2000 nm	-0.06	0.06	-0.19	0.22	3	20.2	0.02	5	2.56	0.28	10
29	SGCT-P8	Precipitated silica	2500 nm	-0.10	0.04	-0.04	0.19	3	21.3	0.02	5	2.78	0.79	9
30	SGCT-P9	Precipitated silica	3500 nm	-0.01	0.05	0.01	0.06	3	16.9	0.03	5	2.61	0.41	7
31	SGCT-Q1	Quartz	-	-0.27	0.03	-0.58	0.14	3	12.6	0.05	5	-	-	-
32	SGCT-Q2	Quartz	-	-0.15	0.04	-0.54	0.08	3	11.6	0.06	5	-	-	-
33	SHMK-P	Precipitated silica	7631-86-9(CAS), 30 nm, hydrophobic	-0.24	0.05	-0.49	0.09	3	18.1	0.06	5	-	-	-
34	THSL-P1	Precipitated silica	TB-2, hydrophilic	-0.07	0.08	-0.34	0.06	3	14.4	0.05	5	-	-	-
35	THSL-P2	Precipitated silica	T20, 1500nm, hydrophilic	-0.23	0.05	-0.41	0.09	3	13.6	0.06	5	-	-	-
36	THSL-P3	Precipitated silica	general	-0.14	0.00	-0.25	0.07	3	14.7	0.05	5	-	-	-
37	THSL-P4	Precipitated silica	hydrophobic	-0.14	0.07	-0.38	0.08	3	16.1	0.07	5	-	-	-
38	THSL-P5	Precipitated silica	T150, hydrophilic	-0.16	0.03	-0.39	0.08	3	16.4	0.06	5	-	-	-
39	THSL-P6	Precipitated silica	36-5, hydrophilic	-0.18	0.06	-0.53	0.03	3	14.7	0.05	5	-	-	-
40	THSL-Q	Quartz	-	-0.12	0.06	-0.31	0.09	3	11.4	0.04	5	-	-	-
41	Wacker-F1	Fumed silica	Germany, N20, hydrophilic	-0.12	0.10	-0.45	0.13	3	23.0	0.03	5	2.04	0.28	15
42	Wacker-F2	Fumed silica	Germany, H18, hydrophobic	-2.15	0.03	-4.48	0.32	3	20.3	0.04	5	2.12	0.22	12
43	Degussa-F1	Fumed silica	Germany, A200, 12 nm, hydrophilic	-2.11	0.08	-4.56	0.37	3	22.4	0.03	5	2.11	0.16	6
44	Degussa-F2	Fumed silica	Germany, R812, hydrophobic	-0.67	0.05	-1.34	0.01	2	21.8	0.02	5	2.47	0.29	12
45	Aladdin-P	Precipitated silica	S104584	-0.09	0.07	-0.12	0.07	3	19.5	0.07	5	-	-	-

46	Aladdin-F1	Fumed silica	S104587	-2.60	0.06	-5.03	0.09	3	19.6	0.04	5	-	-	-
47	Aladdin-F2	Fumed silica	S104588	-1.90	0.02	-3.87	0.49	3	21.2	0.04	5	-	-	-
48	Aladdin-F3	Fumed silica	S104592	-1.10	0.02	-2.31	0.10	3	20.8	0.04	5	-	-	-
49	Aladdin-F4	Fumed silica	S104590	-0.12	0.06	-0.29	0.12	3	20.8	0.03	5	-	-	-
50	Aladdin-F5	Fumed silica	S104589	-2.82	0.05	-5.54	0.10	3	21.3	0.02	5	-	-	-
51	Aladdin-Q1	Quartz	S140828	0.01	0.01	-0.16	0.11	3	10.8	0.04	5	-	-	-
52	Aladdin-Q2	Quartz	S104577	-0.03	0.03	-0.23	0.02	3	11.9	0.07	5	-	-	-
53	NCPS-S1	Sol-gel silica	50 nm, hydrophilic	-0.58	0.05	-1.26	0.03	3	29.9	0.04	5	-	-	-
54	NCPS-S2	Sol-gel silica	100 nm, hydrophilic	-0.60	0.03	-0.95	0.07	3	23.0	0.03	5	-	-	-
55	NCPS-S3	Sol-gel silica	200 nm, hydrophilic	-0.60	0.03	-1.27	0.09	3	27.3	0.04	5	-	-	-
56	NCPS-S4	Sol-gel silica	400 nm, hydrophilic	-0.60	0.08	-0.97	0.05	3	22.0	0.04	5	-	-	-
57	TJKMO-Q	Quartz	-	-0.17	0.05	-0.33	0.05	3	9.8	0.02	5	2.08	0.20	11
58	TJZY-Q	Quartz	-	-0.04	0.01	0.08	0.06	3	12.3	0.03	5	2.15	0.53	11
59	WXYT-Q	Quartz	-	-0.01	0.01	-0.13	0.03	3	9.8	0.02	5	2.00	0.27	6
60	TJDM-Q	Quartz	-	-0.14	0.07	-0.27	0.08	3	12.1	0.03	5	2.21	0.40	9
61	FYTF-Q	Quartz	-	-0.14	0.04	-0.10	0.21	3	12.3	0.02	5	1.42	0.56	6
62	TJFC-D	Diatomite	-	0.06	0.01	0.36	0.14	3	24.0	0.03	5	2.06	0.65	10
63	TJHX-D	Diatomite	-	0.15	0.01	0.29	0.05	3	22.7	0.03	5	1.71	0.36	5
64	JLDH-D1	Diatomite	-	0.13	0.05	0.25	0.11	3	19.0	0.04	5	-	-	-
65	JLDH-D2	Diatomite	-	0.03	0.04	0.15	0.07	3	18.6	0.04	5	-	-	-
66	IFM-1^b	Inorganic filter membrane	-	-0.36	0.02	-0.67	0.09	3	15.0	0.04	5	-	-	-
67	IFM-2^b	Inorganic filter membrane	-	-0.35	0.01	-0.72	0.06	3	14.3	0.06	5	-	-	-
68	IFM-3^b	Inorganic filter membrane	-	-0.25	0.06	-0.62	0.03	3	14.9	0.02	5	-	-	-
69	TP-1^b	Toothpaste	Liangmianzhen,	0.08	0.06	-0.09	0.05	3	19.6	0.04	5	-	-	-

			Freshing particles										
70	TP-2[b]	Toothpaste	Aekyung, 2080	-0.27	0.11	-0.6	0.14	3	21.4	0.02	5	-	-	-
71	TP-3[b]	Toothpaste	Pororo, for kids	-0.11	0.13	-0.34	0.02	3	22.6	0.04	5	-	-	-
72	TP-4[b]	Toothpaste	Cellamiss, Dazzle series	-0.42	0.07	-0.92	0.06	3	23.5	0.04	5	-	-	-
73	NQC[b]	Nanoquartz coating	MagicGem	-0.46	0.06	-0.84	0.13	3	2.1	0.02	5	-	-	-

[a] The nomenclature of sample is “abbreviation of manufacturer + type + sample number”. The abbreviation of manufacturers are given in Supplementary Table 2. The type letter P, S, F, Q, and D denote precipitated silica, sol-gel silica, fumed silica, quartz, and diatomite, respectively.

[b] The #66-73 samples are real consumer products. IFM, TP, and NQC represent inorganic filter membrane, toothpaste, and nanoquartz coating.

Supplementary Table 2. Detailed information for manufacturers or suppliers of samples used in this study.

No.	Abbreviation[a]	Full name	Location	Website
1	WFSJ	Weifang Sanjia Chemical Co.	China	http://www.wfsanjia.com/Product/EYHG/
2	TCTS	Tangshan Caofeidian Taiheng Shengda New Materials Co.	China	http://www.thsdhn.com/
3	BJDK	Beijing DK Nano Technology Co.	China	http://www.nanoinglobal.com/
4	XCJR	Xuancheng Jing Rui New Material Co.	China	http://www.jingruinano.com/
5	SGCT	Shouguang Chang Tai Micro-Nano Chemical Plant	China	http://www.sgchangtai.cn/
6	SHMK	Shanghai Maikun Chemical Co.	China	https://shmaikun.1688.com/
7	THSL	Tonghua Shuanglong Chemical Co.	China	http://www.thdoubledragon.com/
8	Wacker	Wacker Chemie AG	Germany	https://www.wacker.com/
9	Degussa	Evonik Industries AG	Germany	http://corporate.evonik.cn/
10	Aladdin	Aladdin Co.	China	http://www.aladdin-e.com/
11	NCPS	NanoComposix	USA	https://nanocomposix.com/
12	TJK	Tianjin Kemiou Chemical Reagent Co.	China	http://www.chemreagent.com/
13	TJZY	Tianjin Zhiyuan Chemical Reagent Co.	China	http://www.zhiyuanhx.cn/
14	WXYT	Wuxi Yatai United Chemical Co.	China	not available
15	TJDM	Tianjin Damao Chemical Reagent Factory	China	http://www.dmreagent.com/
16	FYTF	Fengyang Tengfei Sand Co.	China	http://www.fytfsys.com/
17	TJFC	Tianjin Fuchen Chemical Reagent Co.	China	http://www.tjfch.com/
18	TJHX	Tianjin Hengxing Chemical Reagent Co.	China	http://13690197953372.gw.1688.com/?tbpm=3
19	JLDH	Jilin Dahua Diatomite Industrial Co.	China	http://www.ljdhsm.com/
20	IFM-1	Ahlstrom-Munksj ö	Sweden	www.munktell.com
21	IFM-2	Pall Corporation	USA	https://www.pall.com/
22	IFM-3	GE Whatman	UK	http://www.gelifesciences.com.cn/
23	TP-1	Liuzhou Liangmianzhen Co.	China	http://www.lmz.com.cn/
24	TP-2	Aekyung Co.	Korea	http://www.aekyung.co.kr/KR/main/main.do
25	TP-3	Pororo Co.	Korea	not available

26	TP-4	Cellamiss Co.	USA	not available
27	NQC	MagicGem Co.	USA	http://www.magicgem.co.uk/

Supplementary Table 3. The probability of five potential sources (EP / EF / ES / NQ / ND) of SiO_2 NP samples given by the five-class LDA-based classification model.[a]

Sample	Probability of source				
	EP	EF	ES	NQ	ND
EP-1	0.657	0.009	0.315	0.010	0.008
EP-2	0.750	0.006	0.190	0.053	0.001
EP-3	0.772	0.002	0.167	0.056	0.002
EP-4	0.353	0.001	0.305	0.001	0.341
EP-5	0.771	0.002	0.140	0.086	0.001
EP-6	0.749	0.003	0.214	0.030	0.005
EP-7	0.781	0.000	0.168	0.037	0.014
EP-8	0.772	0.002	0.165	0.059	0.002
EP-9	0.778	0.001	0.147	0.071	0.002
EP-10	0.776	0.001	0.149	0.072	0.002
EP-11	0.726	0.002	0.242	0.018	0.012
EP-12	0.787	0.000	0.155	0.040	0.018
EP-13	0.778	0.000	0.170	0.033	0.018
EP-14	0.775	0.000	0.167	0.030	0.027
EP-15	0.664	0.000	0.050	0.285	0.001
EP-16	0.717	0.000	0.063	0.219	0.002
EP-17	0.786	0.000	0.153	0.037	0.024
EP-18	0.788	0.000	0.154	0.042	0.016
EP-19	0.758	0.000	0.174	0.024	0.044
EP-20	0.688	0.000	0.051	0.259	0.002
EP-21	0.799	0.000	0.124	0.066	0.011
EP-22	0.775	0.000	0.112	0.110	0.002
EP-23	0.499	0.000	0.026	0.475	0.000
EP-24	0.445	0.000	0.020	0.535	0.000
EP-25	0.516	0.000	0.027	0.457	0.000
EP-26	0.655	0.000	0.053	0.291	0.001
EP-27	0.681	0.000	0.060	0.259	0.001
EP-28	0.543	0.000	0.035	0.422	0.000
EF-1	0.033	0.651	0.315	0.000	0.000
EF-2	0.026	0.621	0.353	0.000	0.000
EF-3	0.116	0.679	0.205	0.000	0.000
EF-4	0.159	0.728	0.105	0.008	0.000
EF-5	0.083	0.737	0.180	0.000	0.000
EF-6	0.069	0.740	0.191	0.000	0.000
EF-7	0.629	0.001	0.306	0.006	0.058
EF-8	0.051	0.706	0.243	0.000	0.000
EF-9	0.037	0.668	0.294	0.000	0.000
EF-10	0.549	0.052	0.390	0.005	0.004
EF-11	0.045	0.689	0.266	0.000	0.000
EF-12	0.056	0.723	0.220	0.000	0.000
EF-13	0.172	0.629	0.198	0.001	0.000
EF-14	0.767	0.000	0.186	0.027	0.019
EF-15	0.030	0.636	0.334	0.000	0.000
ES-1	0.771	0.002	0.167	0.058	0.002
ES-2	0.783	0.000	0.114	0.100	0.003

ES-3	0.716	0.000	0.070	0.212	0.001
ES-4	0.109	0.080	0.518	0.000	0.293
ES-5	0.562	0.012	0.401	0.004	0.021
ES-6	0.225	0.080	0.591	0.000	0.104
ES-7	0.621	0.011	0.350	0.007	0.011
NQ-1	0.350	0.000	0.006	0.644	0.000
NQ-2	0.372	0.000	0.009	0.619	0.000
NQ-3	0.354	0.000	0.005	0.640	0.000
NQ-4	0.371	0.000	0.011	0.618	0.000
NQ-5	0.374	0.000	0.010	0.616	0.000
NQ-6	0.354	0.000	0.007	0.639	0.000
NQ-7	0.366	0.000	0.010	0.624	0.000
NQ-8	0.365	0.000	0.010	0.625	0.000
NQ-9	0.398	0.000	0.016	0.586	0.000
NQ-10	0.363	0.000	0.011	0.626	0.000
NQ-11	0.358	0.000	0.009	0.633	0.000
NQ-12*	0.364	0.000	0.005	0.631	0.000
NQ-13*	0.359	0.000	0.006	0.636	0.000
NQ-14*	0.357	0.000	0.005	0.638	0.000
NQ-15*	0.358	0.000	0.007	0.635	0.000
NQ-16*	0.364	0.000	0.008	0.629	0.000
NQ-17*	0.356	0.000	0.006	0.638	0.000
NQ-18*	0.349	0.000	0.008	0.643	0.000
NQ-19*	0.334	0.000	0.009	0.657	0.000
NQ-20*	0.356	0.000	0.011	0.633	0.000
ND-1	0.481	0.000	0.161	0.004	0.353
ND-2	0.646	0.000	0.164	0.011	0.179
ND-3	0.791	0.000	0.081	0.112	0.015
ND-4	0.782	0.000	0.079	0.130	0.010
ND-5*	0.066	0.000	0.161	0.000	0.773
ND-6*	0.070	0.000	0.154	0.000	0.776
ND-7*	0.064	0.000	0.167	0.000	0.770
ND-8*	0.047	0.000	0.216	0.000	0.738
ND-9*	0.055	0.000	0.192	0.000	0.753
ND-10*	0.281	0.000	0.095	0.002	0.623
ND-11*	0.121	0.000	0.096	0.000	0.782
ND-12*	0.088	0.000	0.112	0.000	0.799
ND-13*	0.033	0.000	0.276	0.000	0.692
ND-14*	0.315	0.000	0.042	0.008	0.636
ND-15*	0.122	0.000	0.073	0.000	0.805
ND-16*	0.045	0.000	0.221	0.000	0.734
ND-17*	0.140	0.000	0.075	0.000	0.785
ND-18*	0.083	0.000	0.123	0.000	0.794
ND-19*	0.064	0.000	0.164	0.000	0.772
ND-20*	0.605	0.000	0.055	0.044	0.296

[a] The pseudo-samples constructed based on the literature data of δ^{30}Si and δ^{18}O are labeled by asterisks.

Supplementary Table 4. The probability of four potential sources (EP+ES / EF / NQ / ND) of SiO_2 NP samples given by the four-class LDA-based classification model.[a]

Sample	Probability of source			
	EP + ES	EF	NQ	ND
EP-1	0.961	0.010	0.024	0.005
EP-2	0.902	0.008	0.089	0.001
EP-3	0.907	0.002	0.089	0.002
EP-4	0.711	0.001	0.002	0.286
EP-5	0.870	0.002	0.127	0.001
EP-6	0.939	0.003	0.055	0.003
EP-7	0.930	0.000	0.059	0.011
EP-8	0.903	0.002	0.093	0.002
EP-9	0.891	0.001	0.106	0.002
EP-10	0.888	0.002	0.109	0.001
EP-11	0.954	0.002	0.035	0.008
EP-12	0.924	0.000	0.062	0.014
EP-13	0.932	0.000	0.054	0.014
EP-14	0.929	0.000	0.049	0.021
EP-15	0.691	0.000	0.308	0.001
EP-16	0.751	0.000	0.247	0.002
EP-17	0.923	0.000	0.058	0.019
EP-18	0.923	0.000	0.064	0.012
EP-19	0.926	0.000	0.039	0.034
EP-20	0.718	0.000	0.280	0.002
EP-21	0.898	0.000	0.093	0.009
EP-22	0.850	0.001	0.148	0.002
EP-23	0.518	0.000	0.482	0.000
EP-24	0.460	0.000	0.540	0.000
EP-25	0.536	0.000	0.464	0.000
EP-26	0.680	0.000	0.319	0.000
EP-27	0.709	0.000	0.290	0.001
EP-28	0.560	0.000	0.440	0.000
EF-1	0.124	0.875	0.000	0.000
EF-2	0.115	0.885	0.000	0.000
EF-3	0.258	0.740	0.002	0.000
EF-4	0.234	0.746	0.020	0.000
EF-5	0.184	0.814	0.001	0.000
EF-6	0.163	0.836	0.001	0.000
EF-7	0.944	0.001	0.013	0.042
EF-8	0.142	0.857	0.001	0.000
EF-9	0.128	0.871	0.000	0.000
EF-10	0.929	0.053	0.016	0.003
EF-11	0.137	0.862	0.001	0.000
EF-12	0.147	0.852	0.001	0.000
EF-13	0.326	0.669	0.006	0.000
EF-14	0.939	0.000	0.047	0.014
EF-15	0.120	0.880	0.000	0.000
ES-1	0.904	0.002	0.092	0.002
ES-2	0.862	0.000	0.135	0.002

ES-3	0.752	0.000	0.247	0.001
ES-4	0.588	0.084	0.000	0.328
ES-5	0.963	0.012	0.011	0.014
ES-6	0.825	0.082	0.001	0.092
ES-7	0.964	0.011	0.018	0.007
NQ-1	0.327	0.000	0.673	0.000
NQ-2	0.372	0.000	0.628	0.000
NQ-3	0.327	0.000	0.673	0.000
NQ-4	0.373	0.000	0.626	0.000
NQ-5	0.376	0.000	0.624	0.000
NQ-6	0.338	0.000	0.661	0.000
NQ-7	0.366	0.000	0.634	0.000
NQ-8	0.364	0.000	0.636	0.000
NQ-9	0.407	0.000	0.593	0.000
NQ-10	0.363	0.000	0.637	0.000
NQ-11	0.352	0.000	0.647	0.000
NQ-12*	0.345	0.000	0.655	0.000
NQ-13*	0.328	0.000	0.672	0.000
NQ-14*	0.327	0.000	0.673	0.000
NQ-15*	0.349	0.000	0.651	0.000
NQ-16*	0.355	0.000	0.644	0.000
NQ-17*	0.328	0.000	0.672	0.000
NQ-18*	0.319	0.000	0.681	0.000
NQ-19*	0.350	0.000	0.650	0.000
NQ-20*	0.349	0.000	0.651	0.000
ND-1	0.687	0.000	0.009	0.304
ND-2	0.831	0.000	0.020	0.149
ND-3	0.850	0.000	0.135	0.014
ND-4	0.837	0.000	0.155	0.009
ND-5*	0.238	0.000	0.001	0.761
ND-6*	0.217	0.000	0.001	0.783
ND-7*	0.126	0.000	0.000	0.874
ND-8*	0.253	0.000	0.002	0.746
ND-9*	0.157	0.000	0.000	0.843
ND-10*	0.144	0.000	0.000	0.856
ND-11*	0.122	0.000	0.000	0.878
ND-12*	0.121	0.000	0.000	0.879
ND-13*	0.193	0.000	0.000	0.807
ND-14*	0.205	0.000	0.000	0.795
ND-15*	0.216	0.000	0.001	0.783
ND-16*	0.260	0.000	0.003	0.737
ND-17*	0.155	0.000	0.000	0.845
ND-18*	0.109	0.000	0.000	0.891
ND-19*	0.159	0.000	0.000	0.841
ND-20*	0.111	0.000	0.000	0.889

[a] The pseudo-samples constructed based on the literature data of δ^{30}Si and δ^{18}O are labeled by asterisks.

Supplementary Table 5. The probability of three potential sources (EP+ES+EF / NQ / ND) of SiO_2 NP samples given by the three-class LDA-based classification model.[a]

Sample	Probability of source		
	EP + EF + ES	NQ	ND
EP-1	0.963	0.026	0.011
EP-2	0.902	0.096	0.002
EP-3	0.905	0.092	0.003
EP-4	0.625	0.002	0.373
EP-5	0.868	0.131	0.002
EP-6	0.938	0.056	0.006
EP-7	0.928	0.057	0.015
EP-8	0.901	0.096	0.003
EP-9	0.889	0.108	0.003
EP-10	0.886	0.112	0.002
EP-11	0.950	0.036	0.014
EP-12	0.924	0.059	0.017
EP-13	0.930	0.052	0.018
EP-14	0.927	0.047	0.026
EP-15	0.695	0.304	0.001
EP-16	0.757	0.241	0.002
EP-17	0.923	0.055	0.022
EP-18	0.923	0.062	0.016
EP-19	0.922	0.037	0.041
EP-20	0.727	0.272	0.002
EP-21	0.901	0.089	0.011
EP-22	0.850	0.148	0.002
EP-23	0.516	0.484	0.000
EP-24	0.457	0.543	0.000
EP-25	0.535	0.465	0.000
EP-26	0.682	0.318	0.001
EP-27	0.711	0.289	0.001
EP-28	0.559	0.441	0.000
EF-1	0.990	0.010	0.000
EF-2	0.992	0.008	0.000
EF-3	0.987	0.011	0.003
EF-4	0.883	0.117	0.000
EF-5	0.988	0.011	0.001
EF-6	0.990	0.009	0.001
EF-7	0.920	0.013	0.067
EF-8	0.980	0.020	0.000
EF-9	0.993	0.006	0.000
EF-10	0.973	0.019	0.008
EF-11	0.974	0.026	0.000
EF-12	0.986	0.014	0.000
EF-13	0.975	0.024	0.001
EF-14	0.935	0.045	0.020
EF-15	0.990	0.010	0.000
ES-1	0.902	0.095	0.003
ES-2	0.863	0.133	0.003

ES-3	0.754	0.245	0.001
ES-4	0.439	0.000	0.561
ES-5	0.956	0.011	0.033
ES-6	0.751	0.001	0.249
ES-7	0.964	0.019	0.017
NQ-1	0.303	0.697	0.000
NQ-2	0.340	0.660	0.000
NQ-3	0.290	0.710	0.000
NQ-4	0.358	0.642	0.000
NQ-5	0.354	0.646	0.000
NQ-6	0.309	0.691	0.000
NQ-7	0.347	0.653	0.000
NQ-8	0.348	0.652	0.000
NQ-9	0.406	0.594	0.000
NQ-10	0.358	0.642	0.000
NQ-11	0.335	0.665	0.000
NQ-12*	0.296	0.704	0.000
NQ-13*	0.315	0.685	0.000
NQ-14*	0.320	0.680	0.000
NQ-15*	0.298	0.702	0.000
NQ-16*	0.361	0.639	0.000
NQ-17*	0.271	0.729	0.000
NQ-18*	0.335	0.665	0.000
NQ-19*	0.356	0.644	0.000
NQ-20*	0.342	0.658	0.000
ND-1	0.681	0.008	0.311
ND-2	0.831	0.018	0.151
ND-3	0.861	0.126	0.013
ND-4	0.846	0.146	0.008
ND-5*	0.179	0.001	0.820
ND-6*	0.141	0.000	0.859
ND-7*	0.075	0.000	0.925
ND-8*	0.201	0.001	0.798
ND-9*	0.079	0.000	0.921
ND-10*	0.084	0.000	0.916
ND-11*	0.077	0.000	0.923
ND-12*	0.081	0.000	0.919
ND-13*	0.127	0.000	0.873
ND-14*	0.153	0.000	0.847
ND-15*	0.140	0.000	0.860
ND-16*	0.204	0.002	0.794
ND-17*	0.094	0.000	0.906
ND-18*	0.098	0.000	0.902
ND-19*	0.115	0.000	0.885
ND-20*	0.092	0.000	0.908

[a] The pseudo-samples constructed based on the literature data of δ^{30}Si and δ^{18}O are labeled by asterisks.

Supplementary Table 6. Source discrimination results of SiO_2 NPs of known sources into four classes by the machine learning model.

Sample	Total	Source identified[a]				Accuracy
		EP + ES	EF	NQ	ND	
SiO_2 NPs	90	Number of correct: 85[b]				94.4%
└ Engineered NPs	50	49[c]		1[d]		98.0%
└ EP	28	27	0	1	0	96.4%
└ ES	7	7	0	0	0	100.0%
└ EF	15	3	12	0	0	80.0%
└ Natural NPs	40	4[c]		36[d]		90.0%
└ NQ	20	0	0	20	0	100%
└ ND	20	4	0	0	16	80.0%

[a] The probability values for candidate sources are given in Supplementary Table 4, and the statistics in this table was based on the most probable source. Other conditions are the same as in Table 1 in the paper.

[b] The “number of correct” means the number of samples with correct discrimination result between engineered and natural SiO_2 NP.

[c] The total number of engineered SiO_2 NPs identified (EP + EF + ES).

[d] The total number of natural SiO_2 NPs identified (NQ + ND).

Supplementary Table 7. Source discrimination results of SiO_2 NPs of known sources into three classes by the machine learning model.

Sample	Total	Source identified[a]			Accuracy
		EP + EF + ES	NQ	ND	
SiO_2 NPs	90	Number of correct: 84[b]			93.3%
└ Engineered NPs	50	48[c]	2[d]		96.0%
└ EP	28	27	1	0	96.4%
└ EF	15	15	0	0	100.0%
└ ES	7	6	0	1	85.7%
└ Natural NPs	40	4[c]	36[d]		90.0%
└ NQ	20	0	20	0	100.0%
└ ND	20	4	0	16	80.0%

[a] The probability values for candidate sources are given in Supplementary Table 5, and the statistics in this table was based on the most probable source. Other conditions are the same as in Table 1 in the paper.

[b] The "number of correct" means the number of samples with correct discrimination result between engineered and natural SiO_2 NP.

[c] The total number of engineered SiO_2 NPs identified (EP + EF + ES).

[d] The total number of natural SiO_2 NPs identified (NQ + ND).

Supplementary Table 8. Raw materials and chemical reactions in industrial synthesis of engineered SiO_2 NPs (see Fig. 3 in the paper).

No.[a]	Raw material	Equation
1, 2	silica or diatomite, powdered carbon, chlorine	$SiO_2 + 2C + 2Cl_2 \xrightarrow{high\ temperature} SiCl_4 + 2CO$
3	silica, coke, steel scrap	$SiO_2 + C + Fe \xrightarrow{high\ temperature} Fe_xSi_y + CO$
4	silica, coke	$SiO_2 + 2C \xrightarrow{high\ temperature} Si + 2CO$
5	silica, sodium hydroxide	$SiO_2 + 2NaOH \xrightarrow{high\ temperature} Na_2SiO_3 + H_2O$
6	ferrosilicon, chlorine	$Fe_xSi_y + Cl_2 \xrightarrow{high\ temperature} SiCl_4 + Fe$
7	industrial silicon, chlorine	$Si + HCl \xrightarrow{\Delta,\ catalyst} SiCl_4 + H_2$
8	industrial silicon, alcohol	$Si + C_2H_5OH \xrightarrow{\Delta,\ catalyst} Si(OC_2H_5)_4 + 2H_2$
9	silicon tetrachloride, alcohol	$SiCl_4 + 4C_2H_5OH \xrightarrow{\Delta,\ catalyst} Si(OC_2H_5)_4 + 4HCl$
10	silicon tetrachloride, hydrogen, oxygen	$2H_2 + O_2 + SiCl_4 \xrightarrow{high\ temperature} SiO_2 + 2H_2O$
11	TEOS, alkali	$Si(OC_2H_5)_4 + 2H_2O \xrightarrow{catalyst} SiO_2 + 4C_2H_5OH$
12	sodium silicate, hydrochloric acid	$Na_2SiO_3 + 2HCl \rightarrow SiO_2 + H_2O + 2NaCl$

[a] The numbers correspond to those in Fig. 3 in the paper.

Supplementary Table 9. The components of extracts from consumer products.[a]

TP-1		TP-2		TP-3		TP-4		IFM-1		IFM-2		IFM-3		NQC	
Component	wt%	Component	wt%	Component	wt%	Component	wt%	Component	wt%	Component	wt%	Component	wt%	Component	wt%
SiO_2	96.97	SiO_2	99.81	SiO_2	99.66	SiO_2	98.57	SiO_2	98.29	SiO_2	99.71	SiO_2	97.96	Si	97.3
TiO_2	2.79	MgO	0.078	Al_2O_3	0.138	TiO_2	1.11	F	0.490	ZnO	0.151	Na_2O	1.06	Ag	2.06
MgO	0.074	SO_3	0.044	MgO	0.082	Al_2O_3	0.104	MgO	0.075	MgO	0.081	CaO	0.35	Zn	0.27
Al_2O_3	0.043	Na_2O	0.020	Na_2O	0.033	MgO	0.076	ZnO	0.066	SO_3	0.012	MgO	0.28	Ti	0.17
SO_3	0.039	Fe_2O_3	0.015	SO_3	0.031	Na_2O	0.056	SO_3	0.021	CaO	0.010	ZnO	0.11	Mg	0.12

[a] The major components of TP and IFM samples were measured by X-ray fluoroscopy (XRF) and the mass fractions were calculated based on the oxide content. The major components of NQC samples were measured by ICP-MS and the mass fractions were calculated based on elemental contents. Only the five highest components are shown in this table.

Supplementary Table 10. The probability of five potential sources (EP / EF / ES / NQ / ND) of SiO_2 NPs in consumer products given by the five-class LDA-based classification model.

Sample	Probability of source				
	EP	EF	ES	NQ	ND
TP-1	0.799	0.000	0.124	0.064	0.013
TP-2	0.710	0.002	0.258	0.014	0.016
TP-3	0.666	0.001	0.269	0.008	0.056
TP-4	0.533	0.012	0.422	0.003	0.030
IFM-1	0.584	0.000	0.045	0.371	0.000
IFM-2	0.525	0.000	0.035	0.439	0.000
IFM-3	0.570	0.000	0.041	0.389	0.000
NQC	0.403	0.000	0.002	0.595	0.000

Supplementary Table 11. The probability of four potential sources (EP+ES / EF / NQ / ND) of SiO_2 NPs in consumer products given by the four-class LDA-based classification model.

Sample	Probability of source			
	EP + ES	EF	NQ	ND
TP-1	0.900	0.000	0.089	0.010
TP-2	0.958	0.002	0.029	0.011
TP-3	0.941	0.001	0.017	0.041
TP-4	0.960	0.011	0.008	0.021
IFM-1	0.601	0.001	0.399	0.000
IFM-2	0.540	0.001	0.460	0.000
IFM-3	0.587	0.000	0.413	0.000
NQC	0.341	0.000	0.659	0.000

Supplementary Table 12. The probability of three potential sources (EP+ES+EF / NQ / ND) of SiO_2 NPs in consumer products given by the three-class LDA-based classification model.

Sample	Probability of source		
	EP + EF + ES	NQ	ND
TP-1	0.903	0.085	0.012
TP-2	0.952	0.029	0.019
TP-3	0.923	0.017	0.061
TP-4	0.944	0.009	0.047
IFM-1	0.599	0.401	0.000
IFM-2	0.539	0.461	0.000
IFM-3	0.585	0.414	0.000
NQC	0.333	0.667	0.000

Supplementary Table 13. Parameters for Si isotope ratio measurement by MC-ICP-MS.

Instrument settings	
RF power	1300 W
Nebulizer pressure	27.6 psi
Hot gas (Ar) flow rate	0.25 L min^{-1}
Membrane gas flow rate	2.68 L min^{-1}
Sample introduction	DeSolvation Nebulizer (DSN-100)<br>PFA nebulizer, uptake rate ~70 µL min^{-1}
Sampler cone (nickel)	“experimental” WA cone (Nu Instruments) for dry mode
Skimmer cone (nickel)	“experimental” WA cone (Nu Instruments) for dry mode
Lens settings	Optimized for maximum analytical signal intensity
Torch	Glass
Collector	L4-^{28}Si, H1-^{29}Si, H6-^{30}Si
Data acquisition parameters	
Scan type	Static measurements
Measurement mode	Medium-resolution with standard-sample-standard bracketing
Measurement intensity (^{28}Si)	3.6 to 7.5 V
Blank signal (pH ~2) HCl of ^{28}Si	20 to 40 mV
Magnet delay time	2 s
Number of blocks	3 block, 10 cycles
Integration time	10 s

4. Supplementary Figures

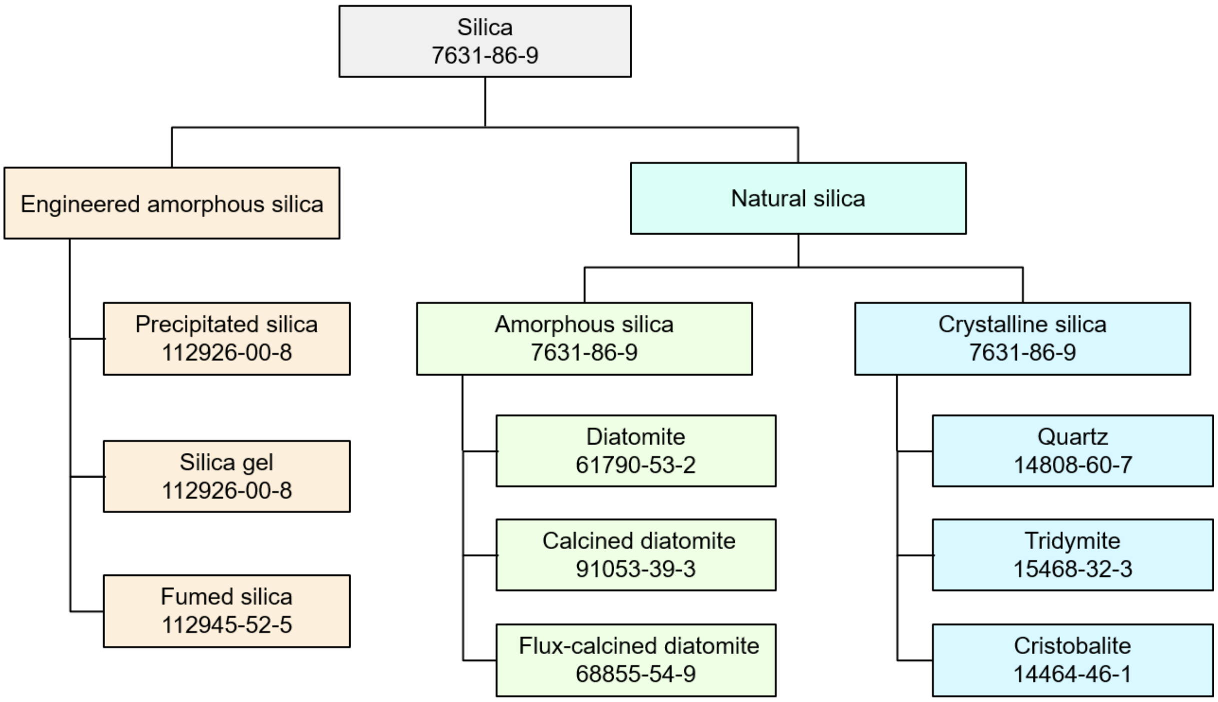

Supplementary Figure 1. CAS numbers of silica in various forms.

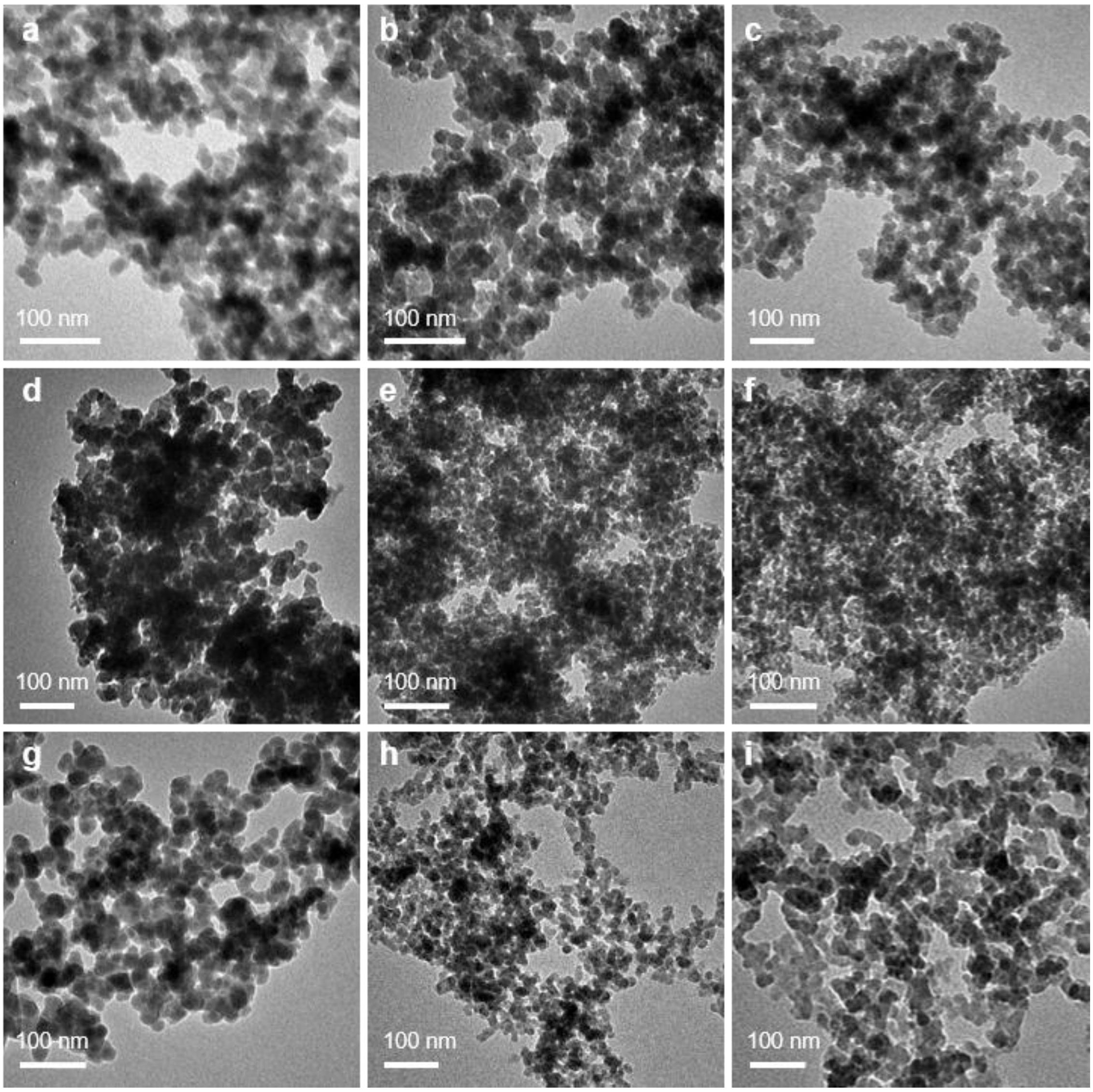

Supplementary Figure 2. TEM measurements of engineered SiO_2 NPs. **a**-**c**, Precipitated silica (EP) from BJDK, XCJR, and SGCT, respectively (see Supplementary Table 2 for detailed information of the suppliers). **d**-**f**, Sol-gel silica (ES) from SGCT. **g**-**i**, Fumed silica (EF) from SGCT, Wacker, and Degussa, respectively. It can be seen that there is no evident difference in shape or aggregation behavior of engineered SiO_2 NPs among different synthetic methods or different manufacturers.

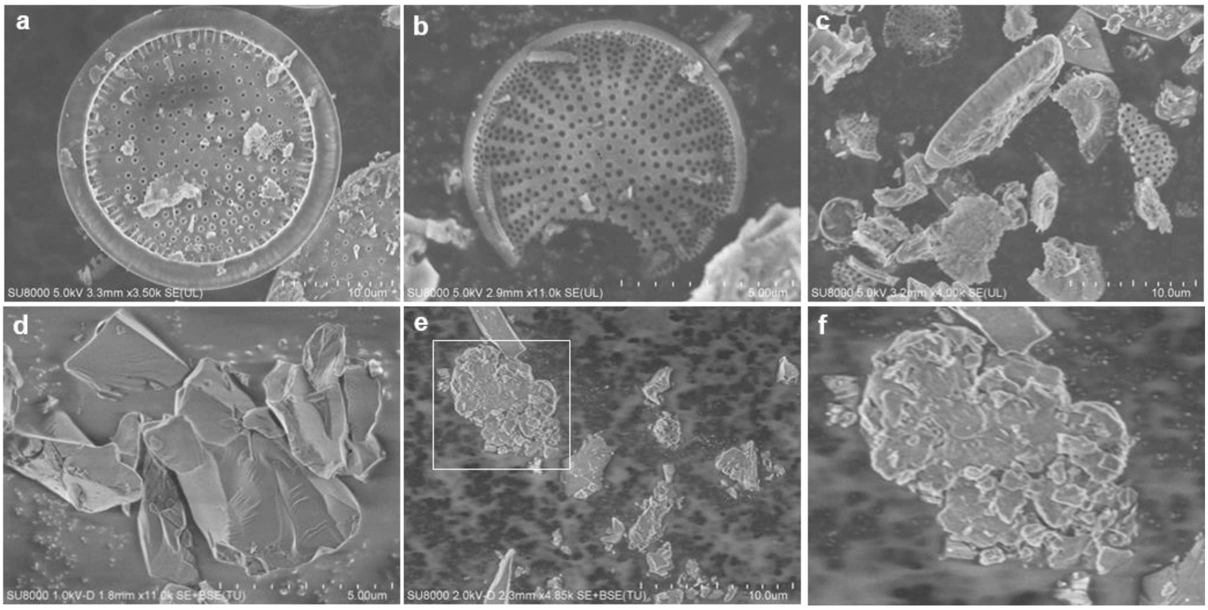

Supplementary Figure 3. SEM measurement showing the different particle morphology in a sample of natural diatomite (**a**-**c**) and quartz (**d**-**f**). **a**-**c**, Intact (**a**), defected (**b**), and fragmentary diatomite particles (**c**). **d**-**e**, Intact (**d**) and fragmentary quartz particles (**e**). **f**, A higher resolution image showing a quartz particle marked in **e**. Although the intact diatomite and quartz particles had characteristic appearance, many defected or unfeatured fragmentary particles were also present in the samples, which might be caused by natural weathering or other physical processes[14]. Therefore, only microscopy measurements are not able to clarify the sources of SiO_2 NPs.

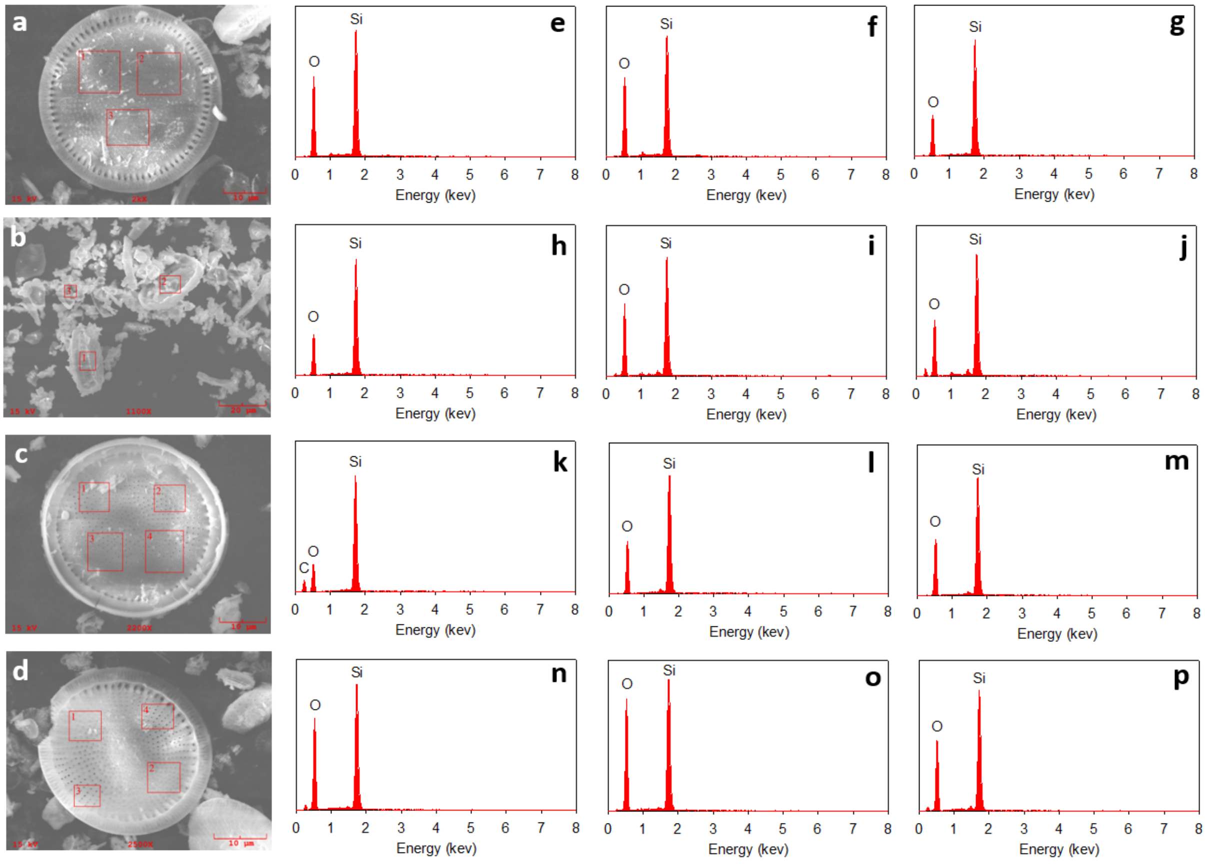

Supplementary Figure 4. SEM and XRD characterization of diatomite. **a-d**, Typical SEM images of diatomite obtained from different suppliers (TJFC (**a**), TJHX (**b**), JLDH (**c**), and JLDH (**d**); see Supplementary Table 2 for detailed information of the suppliers). **e-p**, The XRD patterns obtained at the marked sites in **a**, **b**, **c**, and **d** are shown in **e-g**, **h-j**, **k-m**, and **n-p**, respectively. It can be seen that all diatomite samples have a high purity consisting of only Si and O, but the $R_{O/Si}$ at different locations showed large variations.

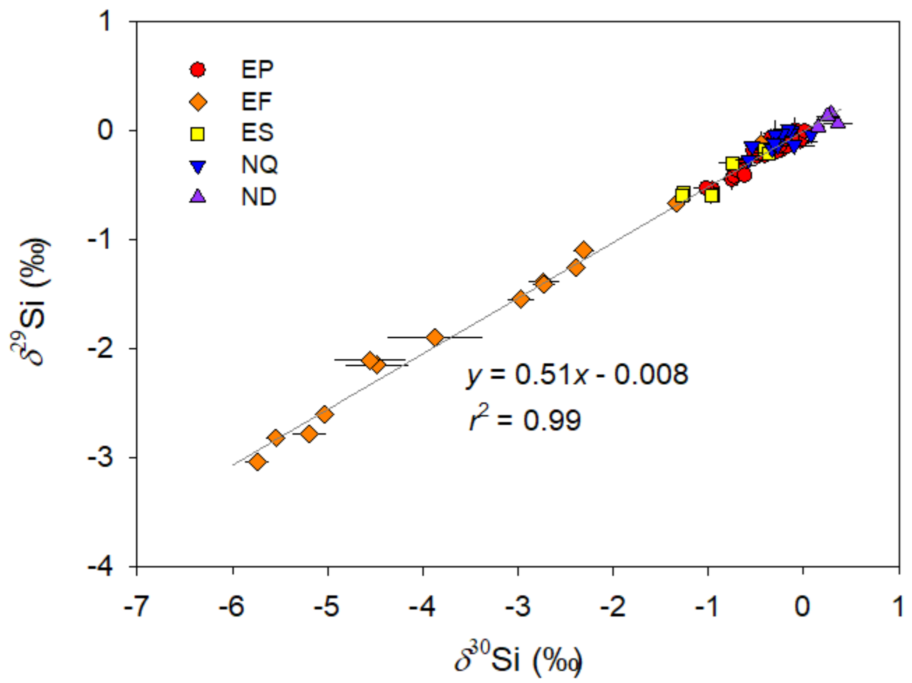

Supplementary Figure 5. Three-isotope plot showing δ^{29}Si versus δ^{30}Si for all SiO_2 NP samples. The solid line represents the theoretical mass-dependent isotope fractionation line. The error bars represent 2SD based on multiple measurements (n = 2-5). It can be seen that the Si isotopic compositions of SiO_2 NP samples accorded with the mass-dependent isotope fractionation.

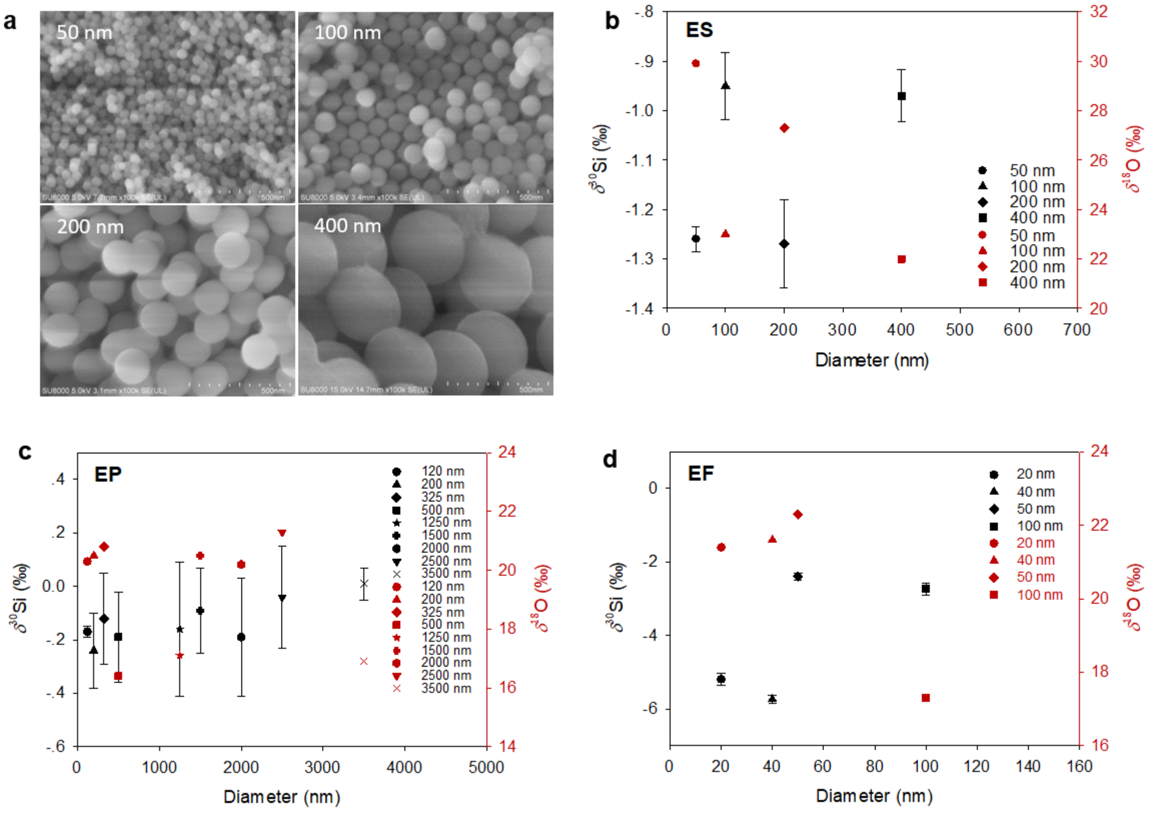

Supplementary Figure 6. Si-O isotopic fingerprints of engineered SiO_2 NPs with different particle sizes. **a**, SEM image of SiO_2 NPs from NCPS with particle size ranging from 50 to 400 nm. **b-d**, Si-O isotopic fingerprints of SiO_2 NPs with different particle sizes synthesized by different methods (ES (**b**), EP (**c**), and EF (**d**)). For a certain method, the samples were from a same manufacturer. The error bars in **b-d** represent 2SD based on multiple measurements (*n* = 2-5). For ES and EP, the δ^{30}Si showed less variations than δ^{18}O; while for EF, the δ^{30}Si also showed a wide variation range. These results were consistent with those in Fig. 2a and 2c. In addition, the O in engineered SiO_2 NPs had more complex sources than the Si (Fig. 3), which might also result in a wider δ^{18}O range than δ^{30}Si. However, for all synthetic methods, the Si-O isotopic fingerprints of engineered SiO_2 NPs displayed no clear trends with their particle size.

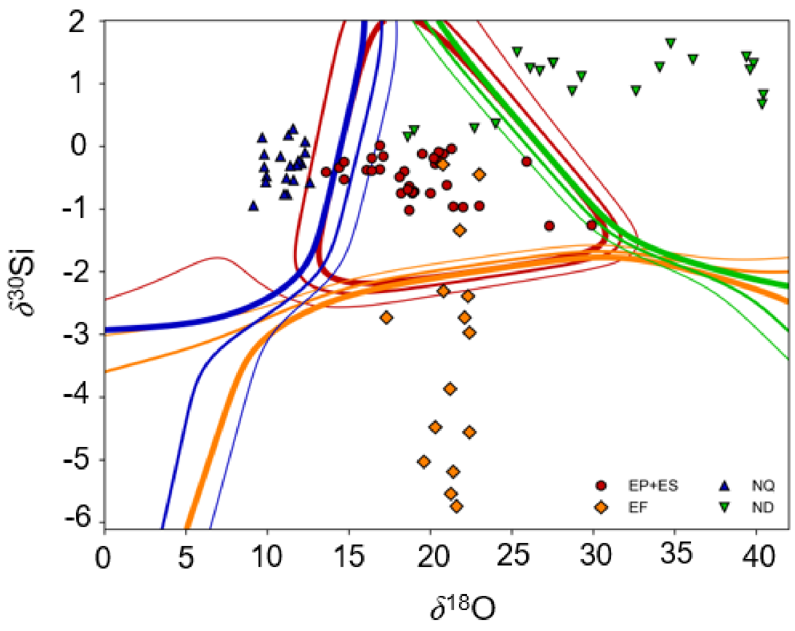

Supplementary Figure 7. Si-O 2D isotopic fingerprints SiO_2 NPs with LDA into four classes (ND / NQ / EF / EP+ES). The zones defined by colored contour lines representing virtual distribution ranges of different sources given by the linear discriminant analysis (LDA)-based classifier. The color and thickness of the contour lines correspond to the respective sources and the probabilities of a sample being predicted to be the related class (0.5, 0.4, 0.3 for the thick, normal, and the thin one, respectively). Other conditions are the same as in Fig. 2 in the paper. The source discrimination results are given in Supplementary Table 4 and 6.

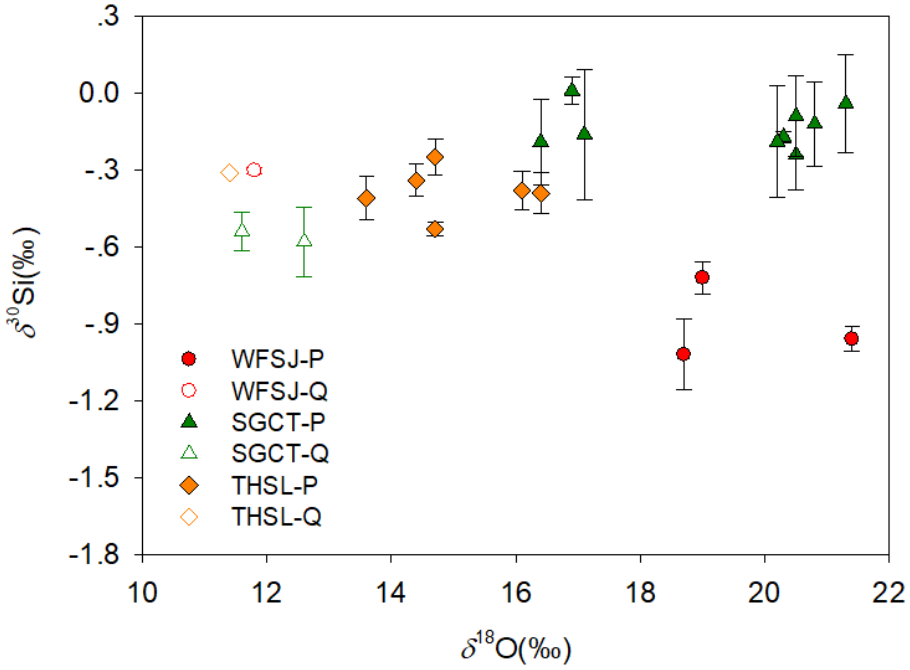

Supplementary Figure 8. Si-O isotopic fingerprints of precipitated SiO_2 NPs (EP) and their raw materials (quartz; NQ) from three different manufacturers. The error bars represent 2SD based on parallel measurements (n = 2-5). The dots in red, orange, and green represent materials from WFSJ (EP-1 and NQ-1), SGCT (EP-2 and NQ-2), and THSL (EP-3 and NQ-3), respectively, and the solid and hollow dots represent the products (EP) and the corresponding raw materials (NQ), respectively. The detailed discussion is given in Supplementary Section 2.2.

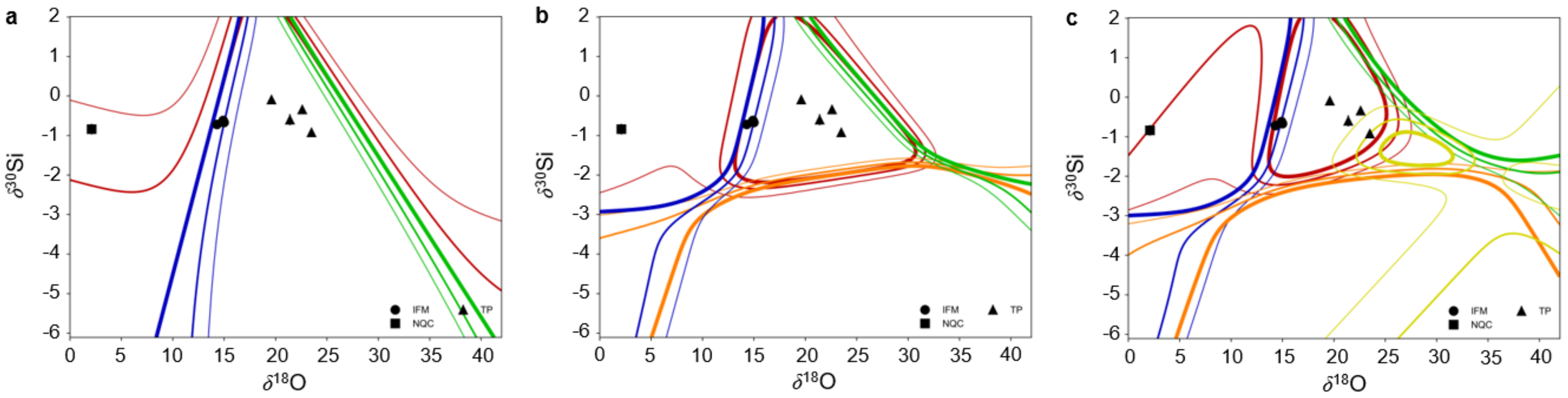

Supplementary Figure 9. Identification of the sources of SiO_2 NPs in consumer products (IFM, TP, and NQC) by Si-O 2D isotopic fingerprints and machine learning model. The zones defined by colored contour lines represent virtual distribution ranges of different sources given by the three- (**a**), four- (**b**), and five-class (**c**) LDA-based classifiers. The color and thickness of the contour lines correspond to the respective sources (the same as in Fig. 2**)** and the probabilities of a sample being predicted to be the related class (0.5, 0.4, 0.3 for the thick, normal, and the thin one, respectively). The probability of the sources of SiO_2 NPs in consumer products are given in Supplementary Table 10-12. From **b** and **c**, because EP and ES could not be well differentiated, the SiO_2 NPs in TP samples were highly probable to originate from EP or ES (probability 90.0–96.0% for EP + ES in four-class LDA; see Supplementary Table 11). This result accorded with the fact that precipitated silica is commonly used as an abrasive and thickening agent in toothpastes due to its abrasive nature. For IFM samples, all data points were distributed at the boundary between NQ and NP. Thus, the SiO_2 NPs in IFM samples probably originated from EP or NQ (probability 52.5–58.4% for EP and 37.1–43.9% for NQ in five-class LDA; see Supplementary Table 10). For the NQC sample, from **a-c**, the SiO_2 NPs most probably came from NQ (probability >59.5% with all three classifiers; see Supplementary Table 10-12), which was consistent with the production description provided by the manufacturer.

5. Supplementary References

1 Barker, P. A. *et al.* A 14,000-year oxygen isotope record from diatom silica in two alpine lakes on Mt. Kenya. *Science* **292**, 2307-2310 (2001).

2 Rietti-Shati, M., Shemesh, A. & Karlen, W. A 3000-year climatic record from biogenic silica oxygen isotopes in an equatorial high-altitude lake. *Science* **281**, 980-982 (1998).

3 Basile-Doelsch, I., Meunier, J. D. & Parron, C. Another continental pool in the terrestrial silicon cycle. *Nature* **433**, 399 (2005).

4 Hu, F. S. & Shemesh, A. A biogenic-silica $\delta^{18}O$ record of climatic change during the last glacial–interglacial transition in southwestern Alaska. *Quatern. Res.* **59**, 379-385 (2003).

5 Varela, D. E., Pride, C. J. & Brzezinski, M. A. Biological fractionation of silicon isotopes in Southern Ocean surface waters. *Global Biogeochem. Cy.* **18** (2004).

6 Douthitt, C. The geochemistry of the stable isotopes of silicon. *Geochim. Cosmochim. Acta* **46**, 1449-1458 (1982).

7 Reynolds, J. H. & Verhoogen, J. Natural variations in the isotopic constitution of silicon. *Geochim. Cosmochim. Acta* **3**, 224-234 (1953).

8 Jun, L. L. New approach to surface seawater palaeotemperatures using $^{18}O/^{16}O$ ratios in silica of diatom frustules. *Nature* **248**, 40 (1974).

9 Clayton, R. N., O'Neil, J. R. & Mayeda, T. K. Oxygen isotope exchange between quartz and water. *J. Geophys. Res.* **77**, 3057-3067 (1972).

10 Matsuhisa, Y., Goldsmith, J. R. & Clayton, R. N. Oxygen isotopic fractionation in the system quartz-albite-anorthite-water. *Geochim. Cosmochim. Acta* **43**, 1131-1140 (1979).

11 De La Rocha, C., Brzezinski, M. A., DeNiro, M. & Shemesh, A. Silicon-isotope composition of diatoms as an indicator of past oceanic change. *Nature* **395**, 680 (1998).

12 Leclerc, A. J. & Labeyrie, L. Temperature dependence of the oxygen isotopic fractionation between diatom silica and water. *Earth Planet. Sci. Lett.* **84**, 69-74 (1987).

13 Wiederhold, J. G. Metal stable isotope signatures as tracers in environmental geochemistry. *Environ. Sci. Technol.* **49**, 2606-2624 (2015).

14 Gaillardet, J., Dupré, B., Louvat, P. & Allegre, C. Global silicate weathering and CO_2 consumption rates deduced from the chemistry of large rivers. *Chem. Geol.* **159**, 3-30 (1999).
